# Supplementary figures and images for: Fibrillar Aβ triggers microglial proteome alterations and dysfunction in Alzheimer mouse models
Source: eLife. 2020 Jun 8;9:e54083. doi: 10.7554/eLife.54083 (PMC7279888; doi:10.7554/eLife.54083)

**A**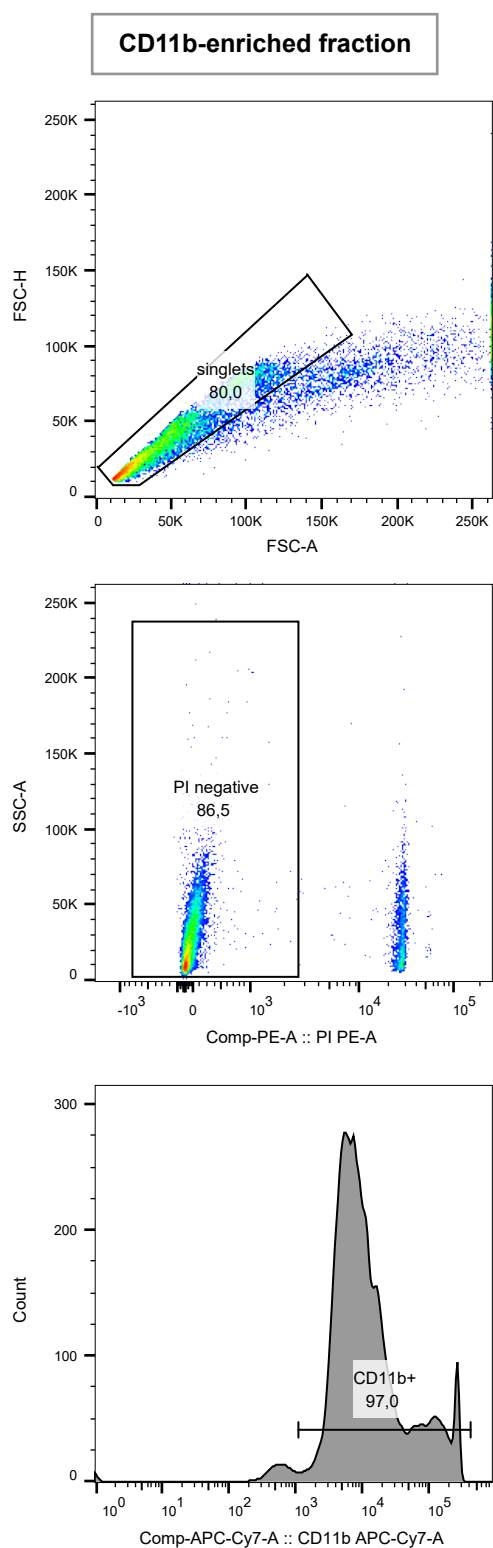**B**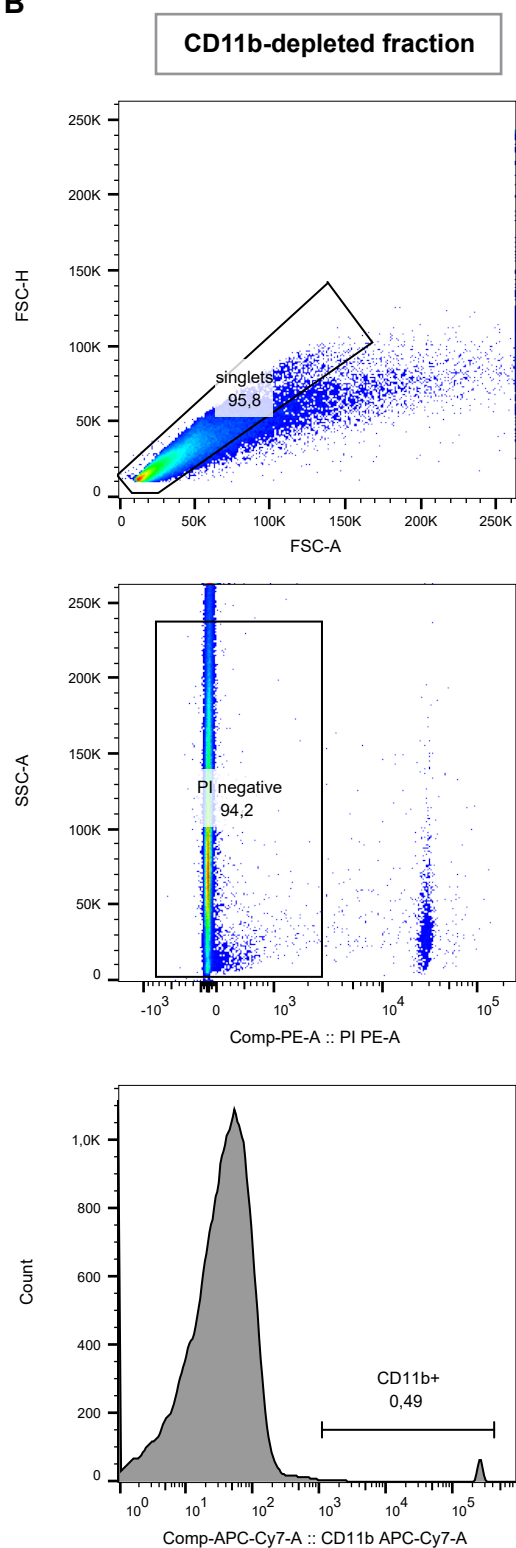

Figure 1-source data 1

Supplement: Figure 1—source data 1. — FACS analysis of the CD11b-enriched (A) and CD11b-depleted fraction (B). Propidium Iodide (PI) was used to analyze cell viability (PI negative fraction). [file elife-54083-fig1-data1.pdf]

**A**

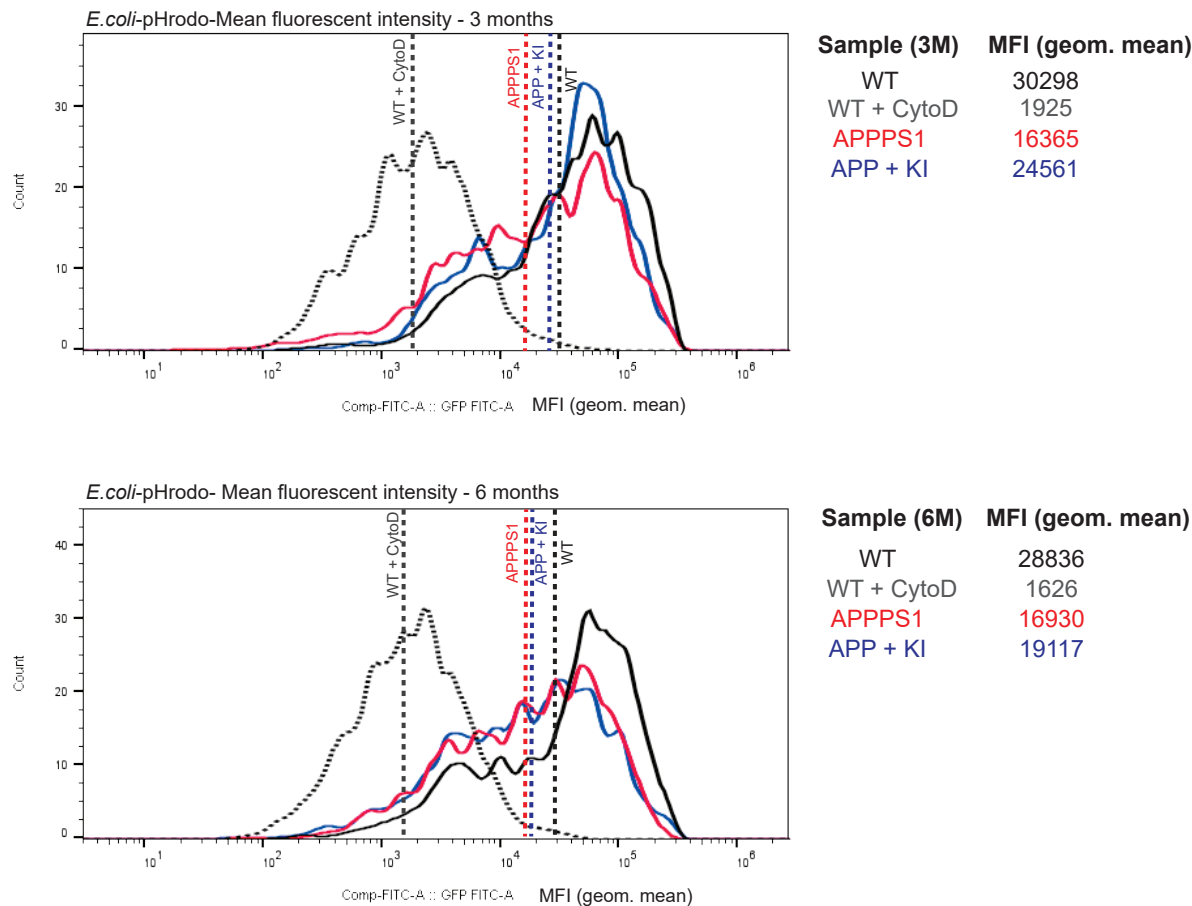

**B**

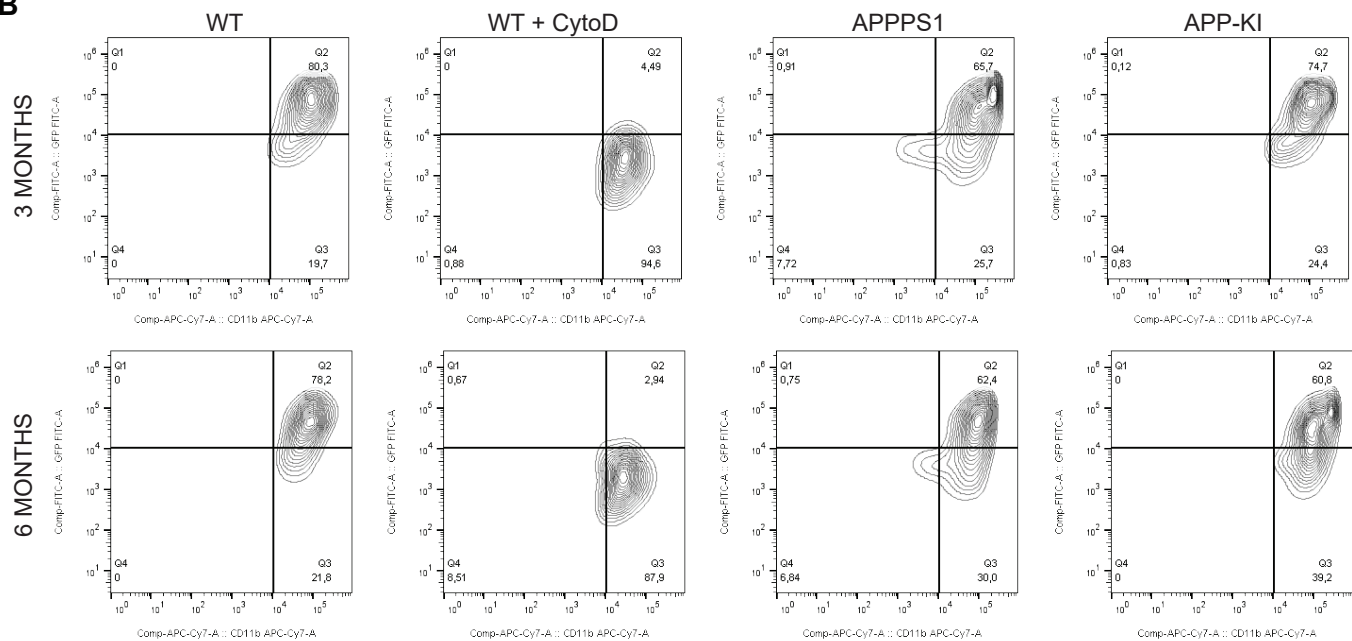

Figure 9-source data 1

Supplement: Figure 9—source data 1. — (A) Histograms represent the fluorescence intensity of uptaken E. coli-pHrodo green particles within the CD11b positive population for every genotype and time point (3 and 6 months). Dashed lines indicate the mean fluorescence intensity (MFI) calculated with the geometric mean for each condition. (B) FACS analysis representing the percentage of CD11b and E. coli-pHrodo positive cells out of the whole CD11b positive population for every genotype. [file elife-54083-fig9-data1.pdf]
