# Supplementary material for: Fibrillar Aβ triggers microglial proteome alterations and dysfunction in Alzheimer mouse models
Source: eLife. 2020 Jun 8;9:e54083. doi: 10.7554/eLife.54083 (PMC7279888; doi:10.7554/eLife.54083)
Supplement: Supplementary file 1. [file elife-54083-supp1.docx]

|  | **DDA** | **DIA** | **DIA vs DDA** |
| --- | --- | --- | --- |
| **Overall Average Peptide IDs** | 53912 | 74281 | 137.8% |
| **Overall Average Protein IDs** | 5502 | 5953 | 108.2% |
| **Overall Average Protein Quantifications** | 5053 | 5952 | 117.8% |
| **Relative quantifications (3 vs 3) APPPS1 vs WT 1M** | 4425 | 5491 | 124.1% |
| **Relative quantifications (3 vs 3) APPPS1 vs WT 3M** | 4646 | 5789 | 124.6% |
| **Relative quantifications (3 vs 3) APPPS1 vs WT 6M** | 4391 | 5848 | 133.2% |
| **Relative quantifications (3 vs 3) APPPS1 vs WT 12M** | 4185 | 5669 | 135.5% |
| **Average of relative quantifications** | 4412 | 5699 | 129.3% |

**Supplementary file 1**
