# Supplementary material for: Fibrillar Aβ triggers microglial proteome alterations and dysfunction in Alzheimer mouse models
Source: eLife. 2020 Jun 8;9:e54083. doi: 10.7554/eLife.54083 (PMC7279888; doi:10.7554/eLife.54083)
Supplement: Supplementary file 2. — The table shows the number of consistently quantified proteins as well as proteins with a significant up- or down-regulation with and without FDR correction. A log2 fold change >0.5 or<−0.5 and a p-value of less than 0.05 were applied as regulation thresholds. The amount of up-and down-regulated proteins with FDR correction is shown as percentage from the total number of quantified proteins. [file elife-54083-supp2.docx]

**A**

|  | **APPPS1**  **vs**  **WT**  **1M** | **APPPS1**  **vs**  **WT**  **3M** | **APPPS1**  **vs**  **WT**  **6M** | **APPPS1**  **vs**  **WT**  **12M** |
| --- | --- | --- | --- | --- |
| **Quantifications (3 vs 3)** | 5491 | 5789 | 5848 | 5669 |
| **Regulated** | 98 | 1010 | 679 | 1409 |
| **Up-regulated** | 76 | 332 | 365 | 776 |
| **Down-regulated** | 22 | 678 | 314 | 633 |
| **Up-regulated FDR corrected** | 0 | 332 | 309 | 776 |
| **Down-regulated FDR corrected** | 0 | 678 | 261 | 633 |
| **Up-regulated FDR corrected (%)** | 0.0% | 5.7% | 5.3% | 13.7% |
| **Down-regulated FDR corrected (%)** | 0.0% | 11.7% | 4.5% | 11.2% |

**B**

|  | **APP-KI**  **vs**  **WT**  **1M** | **APP-KI**  **vs**  **WT**  **3M** | **APP-KI**  **vs**  **WT**  **6M** | **APP-KI**  **vs**  **WT**  **12M** |
| --- | --- | --- | --- | --- |
| **Quantifications (3 vs 3)** | 5713 | 5711 | 5653 | 5715 |
| **Regulated** | 41 | 700 | 559 | 1337 |
| **Up-regulated** | 19 | 22 | 245 | 704 |
| **Down-regulated** | 54 | 109 | 267 | 666 |
| **Up-regulated FDR corrected** | 0 | 1 | 140 | 704 |
| **Down-regulated FDR corrected** | 0 | 0 | 151 | 666 |
| **Up-regulated FDR corrected (%)** | 0.0% | 0.0% | 2.5% | 12.3% |
| **Down-regulated FDR corrected (%)** | 0.0% | 0.0% | 2.7% | 11.7% |

# Supplementary file 2
