# Supplementary material for: Fibrillar Aβ triggers microglial proteome alterations and dysfunction in Alzheimer mouse models
Source: eLife. 2020 Jun 8;9:e54083. doi: 10.7554/eLife.54083 (PMC7279888; doi:10.7554/eLife.54083)
Supplement: Supplementary file 3. [file elife-54083-supp3.docx]

| **Age** | **Genotype** | **Sex per genotype (N=3)** |
| --- | --- | --- |
| 1M | WT | ♂♀♀ |
|  | APPPS1 |  |
| 3M | WT | ♂♂♂ |
|  | APPPS1 |  |
| 6M | WT | ♂♂♀ |
|  | APPPS1 |  |
| 12M | WT | ♂♂♀ |
|  | APPPS1 | ♂♀♀ |
| 1M | WT | ♂♂♀ |
|  | APP-KI |  |
| 3M | WT | ♂♂♀ |
|  | APP-KI |  |
| 6M | WT | ♂♂♀ |
|  | APP-KI |  |
| 12M | WT | ♂♂♀ |
|  | APP-KI |  |

**Supplementary file 3**
