# Supplementary material for: Fibrillar Aβ triggers microglial proteome alterations and dysfunction in Alzheimer mouse models
Source: eLife. 2020 Jun 8;9:e54083. doi: 10.7554/eLife.54083 (PMC7279888; doi:10.7554/eLife.54083)
Supplement: Supplementary file 4. [file elife-54083-supp4.docx]

| **Window** | **m/z start** | **m/z end** | **Center** | **Isolation width [m/z]** |
| --- | --- | --- | --- | --- |
| 1 | 300 | 360 | 330 | 60 |
| 2 | 359 | 399 | 379 | 40 |
| 3 | 398 | 428 | 413 | 30 |
| 4 | 427 | 452 | 439.5 | 25 |
| 5 | 451 | 475 | 463 | 24 |
| 6 | 474 | 497 | 485.5 | 23 |
| 7 | 496 | 518 | 507 | 22 |
| 8 | 517 | 539 | 528 | 22 |
| 9 | 538 | 560 | 549 | 22 |
| 10 | 559 | 581 | 570 | 22 |
| 11 | 580 | 602 | 591 | 22 |
| 12 | 601 | 623 | 612 | 22 |
| 13 | 622 | 646 | 634 | 24 |
| 14 | 645 | 669 | 657 | 24 |
| 15 | 668 | 694 | 681 | 26 |
| 16 | 693 | 719 | 706 | 26 |
| 17 | 718 | 746 | 732 | 28 |
| 18 | 745 | 777 | 761 | 32 |
| 19 | 776 | 808 | 792 | 32 |
| 20 | 807 | 839 | 823 | 32 |
| 21 | 838 | 870 | 854 | 32 |
| 22 | 869 | 904 | 886.5 | 35 |
| 23 | 903 | 943 | 923 | 40 |
| 24 | 942 | 1,122 | 1,032 | 180 |
| 25 | 1,121 | 1,401 | 1,261 | 280 |

# Supplementary file 4
