# Supplementary material for: Fibrillar Aβ triggers microglial proteome alterations and dysfunction in Alzheimer mouse models
Source: eLife. 2020 Jun 8;9:e54083. doi: 10.7554/eLife.54083 (PMC7279888; doi:10.7554/eLife.54083)
Supplement: Supplementary file 5. [file elife-54083-supp5.docx]

| **Experiments** | **Age** | **Genotype** | **Sex** | **Technical replicates** |
| --- | --- | --- | --- | --- |
| - Microglia number/plaque area - Plaque size - Dystrophic neurite size - Microglia number/dystrophic neurite size | 3M | APPPS1 | ♂♀♀♀♀ (N=5) | 30 cortical (neocortex) plaques from 6 brain sections per mouse |
|  |  | APP-KI | ♂♂♂♂ (N=4) |  |
| - CD68 coverage/plaque area - CD68 coverage/microglia number | 3M | APPPS1 | ♂♂♀ (N=3) | 30 cortical (neocortex) plaques from 3 brain sections per mouse |
|  |  | APP-KI | ♂♂♂ (N=3) |  |
| - Total Aβ coverage | 3M | APPPS1 | ♂♀♀♀ (N=4) | 18 cortical (neocortex) images from 3 brain sections per mouse |
|  |  | APP-KI | ♂♂♂♂ (N=4) |  |
| - % pE3-Aβ coverage - % pE3- Aβ coverage/total Aβ coverage | 12M | APPPS1 | ♂♂♂♀ (N=4) |  |
|  |  | APP-KI | ♂♀♀♀ (N=4) |  |
| - LCOs analysis | 3M | APPPS1 | ♀♀♀ (N=3) | 20 cortical (neocortex) plaques from 3 brain sections per mouse |
|  |  | APP-KI | ♂♂♂ (N=3) |  |
|  | 12M | APPPS1 | ♂♂♀ (N=3) |  |
|  |  | APP-KI | ♂♀♀ (N=3) |  |

**Supplementary file 5**
