# Supplementary material for: Fibrillar Aβ triggers microglial proteome alterations and dysfunction in Alzheimer mouse models
Source: eLife. 2020 Jun 8;9:e54083. doi: 10.7554/eLife.54083 (PMC7279888; doi:10.7554/eLife.54083)
Supplement: Supplementary file 6. — Explanation of functions is delineated in green. [file elife-54083-supp6.docx]

n=getDirectory("Choose a Directory to save");

# A

imageCount = nImages

for (image = 1; image <= imageCount; image++) {

name=getTitle();

//print(imageName);

run("Z Project...", "projection=[Max Intensity]"); //Creates a merged image with a maximum intensity projection from the z-stack

run("Split Channels");

blue = "C1-MAX_" + name; //DAPI channel

magenta = "C2-MAX_" + name; //Abeta channel

selectWindow(magenta);//Abeta channel

run("8-bit"); //binary mode

setMinAndMax(8,180); //changes levels of brightness-contrast for this channel

run("Auto Threshold", "method=Otsu white");//threshold filter

setOption("BlackBackground", true);

run("Convert to Mask");

run("Analyze Particles...", "size=10-Infinity show=Outlines display summarize");//only quantifies particles that have an area bigger than 10 µm saveAs("Results",n+name+"abeta"+".xls");//Saves an excel file with the results of all particles quantified and their respective areas

run("Clear Results");

maskcount= "Drawing of " + magenta; //Creates a picture of the area quantified

saveAs("PNG", n+maskcount+"Abeta"+ ".png");//Saves a picture of the area quantified

selectWindow("Summary");

saveAs("Results", n+name + "Abeta-"+"Summary.xls");//Saves an excel file with the sum of the areas of all particles quantified

close();

close();

close();

close();

}

# B

n=getDirectory("Choose a Directory to save");

imageCount = nImages

for (image = 1; image <= imageCount; image++) {

name=getTitle();

//print(imageName);

run("Z Project...", "projection=[Max Intensity]"); //Creates a merged image with a maximum intensity projection from the z-stack

run("Split Channels");

red = "C2-MAX_" + name; //pE3(pyroglu) channel

blue = "C1-MAX_" + name; //DAPI channel

magenta = "C3-MAX_" + name; //Abeta channel

selectWindow(red);//pE3-abeta channel

run("8-bit");//binary mode

setMinAndMax(8,160); //changes levels of brightness-contrast for this channel

run("Threshold...");//to open the threshold window if not opened yet

waitForUser("set the threshold and press OK, or cancel to exit macro");// pauses the execution and lets you set the threshold manually

run("Convert to Mask");

run("Analyze Particles...", "size=2-Infinity show=Outlines display summarize");//only quantifies particles that have an area bigger than 2 µm

saveAs("Results",n+name+"pyroglu"+".xls");//Saves an excel file with the results of all particles quantified and their respective areas

run("Clear Results");//Clears the values quantified from the pE3 channel to re-start recording the ones from the next channel

maskcount= "Drawing of " + red; //Creates a picture of the area quantified

saveAs("PNG", n+maskcount+ "Pyroglu"+ ".png");//Saves a picture of the area quantified

close();

close();

selectWindow(magenta);//Abeta channel

run("8-bit");

setMinAndMax(8,160); //changes levels of brightness-contrast for this channel

run("Threshold...");

waitForUser("set the threshold and press OK, or cancel to exit macro");

run("Convert to Mask");

run("Analyze Particles...", "size=2-Infinity show=Outlines display summarize");//only quantifies particles that have an area bigger than 2 µm

saveAs("Results",n+name+"abeta"+".xls");//Saves an excel file with the results of all particles quantified and their respective areas

run("Clear Results");

maskcount= "Drawing of " + magenta; //Creates a picture of the area quantified

saveAs("PNG", n+maskcount+"Abeta"+ ".png");//Saves a picture of the area quantified

selectWindow("Summary");

saveAs("Results", n+name + "Pyroglu-Abeta-"+"Summary.xls");//Saves an excel file with the sum of the areas of all quantified particles

close();

close();

close();

close();

}

## Supplementary file 6
