# Supplementary material for: Fibrillar Aβ triggers microglial proteome alterations and dysfunction in Alzheimer mouse models
Source: eLife. 2020 Jun 8;9:e54083. doi: 10.7554/eLife.54083 (PMC7279888; doi:10.7554/eLife.54083)
Supplement: Supplementary file 7. [file elife-54083-supp7.docx]

| **Age** | **Genotype** | **Sex** | **Technical replicates** |
| --- | --- | --- | --- |
| 3M | WT | ♂♂♀ (N=3) | 3x *E.coli*,  2x *E.coli* + CytoD |
|  | APPPS1 | ♂♂♂ (N=3) | 4x *E.coli* |
|  | APP-KI | ♂♂♂ (N=3) | 4x *E.coli* |
| 6M | WT | ♀♀ (N=2) | 4x *E.coli*,  2x *E.coli* + CytoD |
|  | APPPS1 | ♂♀ (N=2) | 5x *E.coli* |
|  | APP-KI | ♂♀ (N=2) | 5x *E.coli* |

**Supplementary file 7**
